# Cine MR feature tracking analysis for diagnosing thymic epithelial tumors: a feasibility study

**DOI:** 10.1186/s40644-023-00560-z

**Published:** 2023-05-01

**Authors:** Koji Takumi, Hiroaki Nagano, Akie Mukai, Kazuhiro Ueda, Kazuhiro Tabata, Takashi Yoshiura

**Affiliations:** 1 Department of Radiology, Kagoshima University Graduate School of Medical and Dental Sciences, 8-35-1 Sakuragaoka, Kagoshima City, 890-8544 Japan; 2 General Thoracic Surgery, Kagoshima University Graduate School of Medical and Dental Sciences, 8-35-1 Sakuragaoka, Kagoshima City, 890-8544 Japan; 3 Human Pathology, Kagoshima University Graduate School of Medical and Dental Sciences, 8-35-1 Sakuragaoka, Kagoshima City, 890-8544 Japan

**Keywords:** Thymic epithelial tumors, Thymic cancer, Cine magnetic resonance imaging, Feature tracking

## Abstract

**Background:**

To assess the feasibility of the cine MR feature tracking technique for the evaluation of cardiovascular-induced morphological deformation in the diagnosis of thymic epithelial tumors (TETs).

**Methods:**

Our study population consisted of 43 patients with pathologically proven TETs including 10 low-grade thymomas, 23 high-grade thymomas, and 10 thymic carcinomas. Cine MR images were acquired using a balanced steady-state free precession sequence with short periods of breath-hold in the axial and oblique planes in the slice with the largest lesion cross-sectional area. The tumor margin was manually delineated in the diastolic phase and was automatically tracked for all other cardiac phases. The change rates of the long-to-short diameter ratio (∆LSR) and tumor area (∆area) associated with pulsation were compared between the three pathological groups using the Kruskal–Wallis H test and the Mann–Whitney U test. A receiver-operating characteristic (ROC) curve analysis was performed to assess the ability of each parameter to differentiate thymic carcinomas from thymomas.

**Results:**

∆LSR and ∆area were significantly different among the three groups in the axial plane (p = 0.028 and 0.006, respectively) and in the oblique plane (p = 0.034 and 0.043, respectively). ∆LSR and ∆area values were significantly lower in thymic carcinomas than in thymomas in the axial plane (for both, p = 0.012) and in the oblique plane (p = 0.015 and 0.011, respectively). The area under the ROC curves for ∆LSR and ∆area for the diagnosis of thymic carcinoma ranged from 0.755 to 0.764.

**Conclusions:**

Evaluation of morphological deformation using cine-MR feature tracking analysis can help diagnose histopathological subtypes of TETs and identify thymic carcinomas preoperatively.

**Supplementary Information:**

The online version contains supplementary material available at 10.1186/s40644-023-00560-z.

## Background

Thymic epithelial tumors (TETs) are the most common primary tumors in the anterior mediastinum, with an incidence of approximately 1.3 per million person-years [1]. Clinically, TETs comprise three subgroups according to the WHO classification: low-risk thymomas (type A, AB, and B1), high-risk thymomas (type B2 and B3), and thymic carcinomas. Subgroup is an independent prognostic factor for survival in patients with TETs [2] and is important information for optimizing treatment strategies.

Cine MR imaging is widely used to evaluate cardiovascular diseases by assessment of cardiac morphology and function [3] and can also be used to diagnose chest wall or cardiovascular invasion of thoracic masses by evaluating cardiovascular-induced sliding motion [4–6]. The MR feature tracking technique, a two-dimensional post-processing algorithm based on cine MRI, has recently been introduced into clinical practice. It was developed to evaluate cardiac function and myocardial deformation, which are applied to the diagnosis and prediction of prognosis in cardiac diseases [7]. A recent report [8] showed that MR feature tracking analysis of cardiovascular-induced liver deformation was correlated with liver damage in patients with tetralogy of Fallot. Reduced passive deformity of the liver may be due to chronic liver damage with fibrosis. Therefore, the feature tracking technique may have the potential to provide information on tissue stiffness by evaluation of morphological deformation of a mediastinal lesion (including TETs) that occurs by the pulsation of adjacent cardiovascular structures. The WHO classification of TETs is determined pathologically based on the morphological manifestations of epithelial cells and the ratio of lymphocytes to epithelial cells [9], which can impact tumor stiffness. We hypothesize that evaluation of cardiovascular-induced morphological deformation using cine-MR feature tracking analysis can assist in diagnosing the histopathological classification of TETs. Therefore, the purpose of this study was to assess the diagnostic feasibility of feature tracking analyses using pretreatment cine-MR images for the evaluation of TETs.

## Materials and methods

### Patients

Institutional ethics review board approval was obtained and informed consent was waived for this retrospective study. Between February 2008 and April 2021, all patients who met the following inclusion criteria were enrolled: (a) pathologically confirmed TET, (b) had undergone cine-MR examination, (c) no history of treatment for TET before the MR examination, and (d) lesions larger than 10 mm in short diameter. The final diagnosis was determined by histological examination of biopsy or surgical specimens. The tissues were fixed with 10% neutral phosphate-buffered formalin, routinely processed for paraffin embedding, and sectioned for hematoxylin and eosin (HE) staining. All TET lesions were classified into six histological subtypes according to the 2015 WHO histological classification and divided into the following three subgroups: low-risk thymoma (types A, AB, and B1), high-risk thymoma (types B2 and B3), and thymic carcinoma.

### MR imaging protocol

All MR examinations were performed using 3T systems (Trio, Siemens Healthcare, Erlangen, Germany; or Ingenia 3.0T, Philips Healthcare, Best, The Netherlands) using a 30-channel phased-array body coil during a breath hold. Cine-MR imaging has been a part of our institute’s routine clinical pretreatment MR protocol for the evaluation of chest wall or cardiovascular invasion in anterior mediastinal tumors. Cardiac-gated cine images were acquired using a balanced steady-state free precession (bSSFP) sequence with short periods of breath holding in the axial and oblique planes. The oblique plane was applied perpendicular to the interface between the thymic lesion and the adjacent cardiovascular structures with reference to the axial image. The following parameters were used for imaging in both planes: repetition time, 10 to 48 ms; echo time, 1.5 ms; flip angle, 45 or 50°; turbo field echo factor, 22; number of cardiac phases, 20–25; number of signal averages, 1; field of view, 350 × 350 mm; in-plane spatial resolution, 1 to 2 × 1 to 2 mm; section thickness, 3–6 mm; number of slices, 3–5.

### Imaging analyses

To evaluate cardiac-induced deformation with feature tracking evaluations, all cine images were transferred and analyzed using a workstation (Ziostation 2; Ziosoft Inc., Tokyo, Japan). All images were independently evaluated by a radiologist (with 20 years of chest radiology experience) who was blinded to the final pathological results. All measurements were performed twice by the same observer. The tumor margin was delineated manually in the diastolic phase in the axial and oblique planes on the slice with the largest cross-sectional area of the lesion, and automatically tracked for all other cardiac phases using feature-tracking methods based mainly on a block-matching approach, which defines the region of interest for the target structure and tracks it along the cardiac cycle by searching for the most similar region in the next image [10, 11]. Tumor sizes (longest and shortest diameters) and lesion area were automatically measured for all cardiac phases. We calculated the long-to-short-diameter ratio, which was defined as the short diameter divided by the long diameter. Long diameter (LD_max_ and LD_min_), short diameter (SD_max_ and SD_min_), long-to-short diameter ratio (LSR_max_ and LSR_min_), and lesion area (LA_max_ and LA_min_) were recorded in the two phases when the lesion areas were maximal and minimal, respectively. We also calculated the change in the long-to-short-diameter ratio (∆long-to-short-diameter ratio [∆LSR]) and lesion area (∆area) between the two phases when the lesion areas were maximal and minimal. The length of contact between the lesion and the adjacent cardiovascular structures was measured in both planes.

### Statistical analysis

Intra-observer agreement was assessed by calculating the intra-class correlation coefficient (ICC). ICCs were considered to indicate excellent agreement when > 0.74 [12]. Long diameter (LD_max_ and LD_min_), short diameter (SD_max_ and SD_min_), long-to-short diameter ratio (LSR_max_ and LSR_min_), lesion area (LA_max_ and LA_min_), ∆LSR, and ∆area were compared among the three groups (low- and high-risk thymomas and thymic carcinomas), between low- and high-risk thymomas, and between all thymomas and thymic carcinomas using the Kruskal–Wallis H test or the Mann–Whitney U test. Receiver-operating characteristic (ROC) curve analysis was performed to evaluate the ability of continuous values to differentiate thymic carcinomas from thymomas. Sensitivity and specificity were calculated using a threshold criterion that would maximize the Youden index. All data for continuous variables are presented as mean ± standard deviation. Values of P < 0.05 were considered indicative of significance in all analyses. Statistical analyses were performed using MedCalc version 19.6 (MedCalc Software, Mariakerke, Belgium) and SPSS version 28.0 (SPSS, Chicago, IL).

## Results

### Patients and thymic tumor classifications

**Fig. 1:**
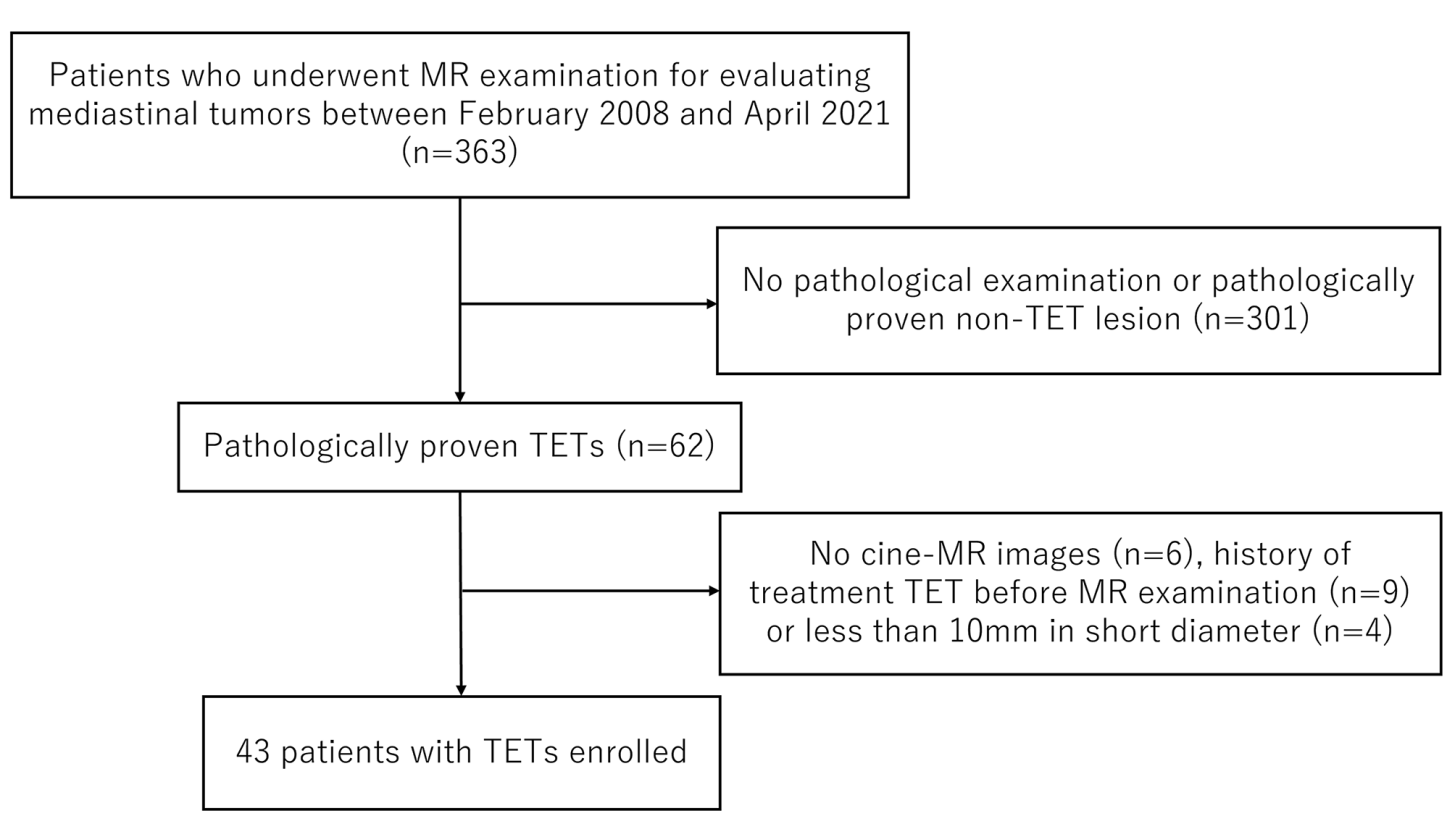
Flow diagram of the study population. Abbreviation: TET = thymic epithelial tumor

Forty-three eligible patients (18 men, 25 women; mean age, 62.3 ± 15.4 years; range, 25–85 years) were identified and included in this study (Fig. 1). The clinical and pathological characteristics of the study population are summarized in Table 1. The subgroups of TETs were diagnosed pathologically as 10 low-risk thymomas (type A [n = 4], AB [n = 3], type B1 [n = 3]), 23 high-risk thymomas (type B2 [n = 8], type B3 [n = 15]), and 10 thymic carcinomas. All thymic tumors were located in the anterior mediastinum.

**Table 1:** Clinical and demographic characteristics

Variables	N = 43
Gender (M: F)	18:25
Age (mean ± SD)	62.3 ± 15.4
Major clinical symptoms	
Pain or pressure in the chest	6
Shortness of breath	1
General fatigue	3
Facial swelling	2
Weight loss	1
Drooping eyelids	9
No symptom	21
Mosaoka-Koga stage	
I	9
II	19
III	9
IV	6
WHO classification	
A	4
AB	3
B1	3
B2	8
B3	15
Carcinoma	10

### Feature tracking parameters among the three TET groups

Intra-observer variability was excellent for all parameters (Online supplementary table). Table 2 lists the values of tracking parameters in the axial and oblique images according to group. There was no significant difference among the groups in terms of contact length between the lesion and adjacent cardiovascular structures in either plane (both p > 0.05). ∆LSR and ∆area were significantly different among the three groups in the axial plane (p = 0.028 and 0.006, respectively) and in the oblique plane (p = 0.034 and 0.043, respectively), whereas there was no significant difference in long diameter (LD_max_ and LD_min_), short diameter (SD_max_ and SD_min_), long-to-short diameter ratio (LSR_max_ and LSR_min_), or lesion area (LA_max_ and LA_min_) between the groups in either plane (all p > 0.05).

**Table 2:** Clinical characteristics and cine MR feature tracking parameters

		Low-risk thymomas (n = 10)	High-risk thymomas (n = 23)	Thymic carcinomas (n = 10)	P (among three groups)	P (Low- vs. high-risk thymomas)	P (all thymomas vs. carcinomas)
**Axial image**	Contact length (mm)	43.2 ± 14.6	33.9 ± 13.5	44.0 ± 16.3	0.101	0.089	0.204
	Long diameter with maximum lesion area (mm)	49.5 ± 16.4	45.2 ± 16.8	52.9 ± 22.9	0.721	0.524	0.702
	Short diameter with maximum lesion area (mm)	30.5 ± 12.0	23.3 ± 9.1	33.5 ± 18.7	0.123	0.105	0.249
	Long-to-short ratio with maximum lesion area	0.6 ± 0.1	0.5 ± 0.1	0.6 ± 0.1	0.193	0.133	0.386
	Long diameter with minimum lesion area (mm)	48.6 ± 16.7	44.7 ± 16.7	52.5 ± 23.0	0.724	0.550	0.661
	Short diameter with minimum lesion area (mm)	28.7 ± 11.5	22.5 ± 9.4	32.9 ± 18.9	0.140	0.155	0.194
	Long-to-short ratio with minimum lesion area	0.6 ± 0.1	0.5 ± 0.2	0.6 ± 0.1	0.241	0.207	0.273
	**∆Long-to-short ratio (%)**	**4.7 ± 3.5**	**4.1 ± 5.8**	**1.4 ± 1.4**	**0.028**	0.253	**0.012**
	Maximum lesion area (mm^2^)	1349.8 ± 918.3	964.7 ± 654.0	1747.0 ± 1942.0	0.294	0.269	0.313
	Minimum lesion area (mm^2^)	1270.0 ± 885.9	929.8 ± 649.0	1717.5 ± 1939.1	0.310	0.305	0.286
	**∆Area (%)**	**6.8 ± 4.2**	**4.9 ± 4.5**	**2.6 ± 1.6**	**0.006**	**0.038**	**0.012**
**Oblique image**	Contact length (mm)	54.1 ± 24.1	43.6 ± 16.2	51.2 ± 23.2	0.524	0.269	0.702
	Long diameter with maximum lesion area (mm)	51.3 ± 19.5	42.6 ± 14.5	53.8 ± 25.7	0.477	0.305	0.487
	Short diameter with maximum lesion area (mm)	30.4 ± 12.5	23.8 ± 10.9	31.7 ± 19.4	0.2829	0.133	0.681
	Long-to-short ratio with maximum lesion area	0.6 ± 0.1	0.6 ± 0.2	0.6 ± 0.1	0.800	0.524	0.788
	Long diameter with minimum lesion area (mm)	50.1 ± 19.5	42.6 ± 14.5	53.3 ± 25.8	0.533	0.343	0.487
	Short diameter with minimum lesion area (mm)	30.4 ± 12.5	23.8 ± 10.9	31.0 ± 18.7	0.266	0.133	0.542
	Long-to-short ratio with minimum lesion area	0.6 ± 0.2	0.6 ± 0.2	0.6 ± 0.1	0.772	0.524	0.810
	**∆Long-to-short ratio (%)**	**6.5 ± 5.9**	**4.6 ± 4.2**	**1.6 ± 1.0**	**0.034**	0.363	**0.015**
	Maximum lesion area (mm^2^)	1339.5 ± 1003.0	950.2 ± 642.4	1704.9 ± 1962.3	0.421	0.305	0.435
	Minimum lesion area (mm^2^)	1336.9 ± 984.0	900.5 ± 637.4	1670.7 ± 1947.7	0.354	0.384	0.356
	**∆Area (%)**	**5.7 ± 3.7**	**6.5 ± 4.8**	**2.8 ± 1.4**	**0.043**	0.862	**0.011**

### Comparison between thymomas and thymic carcinomas

**Fig. 2:**
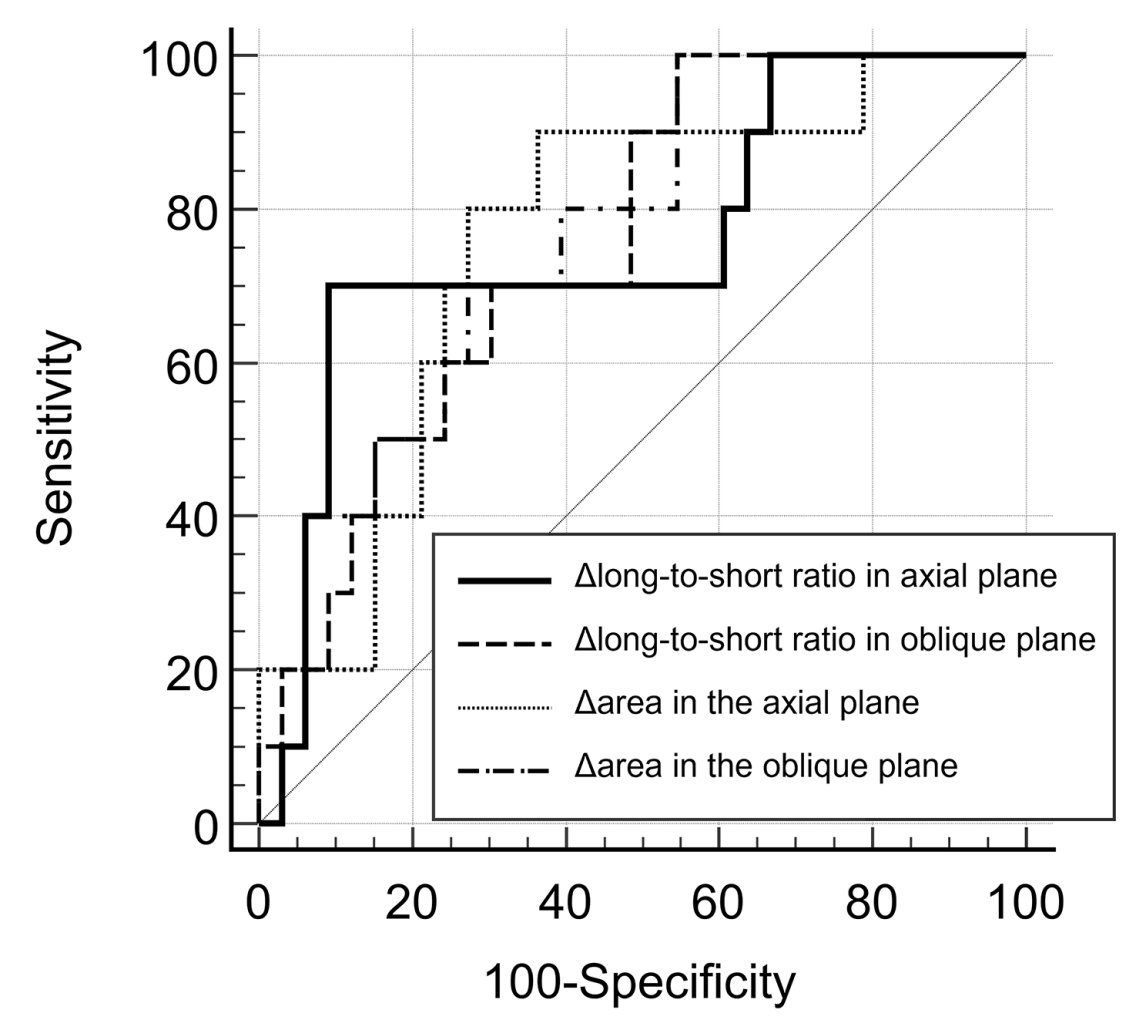
ROC curve analysis of different parameters for diagnosing thymic carcinoma. The area under the ROC curve values were 0.761, 0.755, 0.761, and 0.764 for ∆LSR in the axial and oblique planes, and ∆area in the axial and oblique planes, respectively

The ∆LSR and ∆area of thymic carcinomas were significantly smaller than those of thymomas in the axial plane (4.26 ± 5.13% vs. 1.45 ± 1.42%, p = 0.012; 5.47 ± 4.43% vs. 2.58 ± 1.55%, p = 0.012, respectively) and in the oblique plane (5.19 ± 4.78% vs. 1.65 ± 0.99%, p = 0.015; 6.21 ± 4.43% vs. 2.82 ± 1.41%, p = 0.011, respectively). For diagnosing thymic carcinomas, the values of the area under the ROC curve (AUC) were 0.761 (95% confidential interval [CI], 0.606–0.877), 0.755 (95%CI, 0.600–0.873), 0.761 (95%CI, 0.606–0.877), and 0.764 (95%CI, 0.610–0.880) for ∆LSR in the axial and oblique planes, and ∆area in the axial and oblique planes, respectively (Fig. 2; Table 3). The optimal cutoff values of ∆LSR and ∆area in the axial plane to differentiate thymic carcinoma from thymomas were 0.90% (sensitivity, 70.0% with 95%CI of 34.8–93.3; specificity, 90.9% with 95%CI of 75.7–98.1) and 3.39% (sensitivity, 90% with 95%CI of 55.5–99.7; specificity, 63.6% with 95%CI of 45.1–79.6), respectively (Table 3). The optimal cutoff values of ∆LSR and ∆area in the oblique plane to differentiate thymic carcinoma from thymomas were 3.53% (sensitivity, 100.0% with 95%CI of 69.2–100.0; specificity, 45.5% with 95%CI of 28.1–63.6) and 5.04% (sensitivity, 100% with 95%CI of 69.2–100.0; specificity, 45.5% with 95%CI of 28.1–63.6), respectively (Table 3).

**Table 3:** Area under the ROC curve values for the diagnosis of thymic carcinoma

Parameters	AUC	Threshold Value	Sensitivity (%)	Specificity (%)
∆Long-to-short ratio in the axial plane	0.761	≤ 0.90	70.0	90.9
∆Long-to-short ratio in the oblique plane	0.755	≤ 3.53	100.0	45.5
∆Area in the axial plane	0.761	≤ 3.39	90.0	63.6
∆Area in the oblique plane	0.764	≤ 5.04	100.0	45.5

**Fig. 3:**
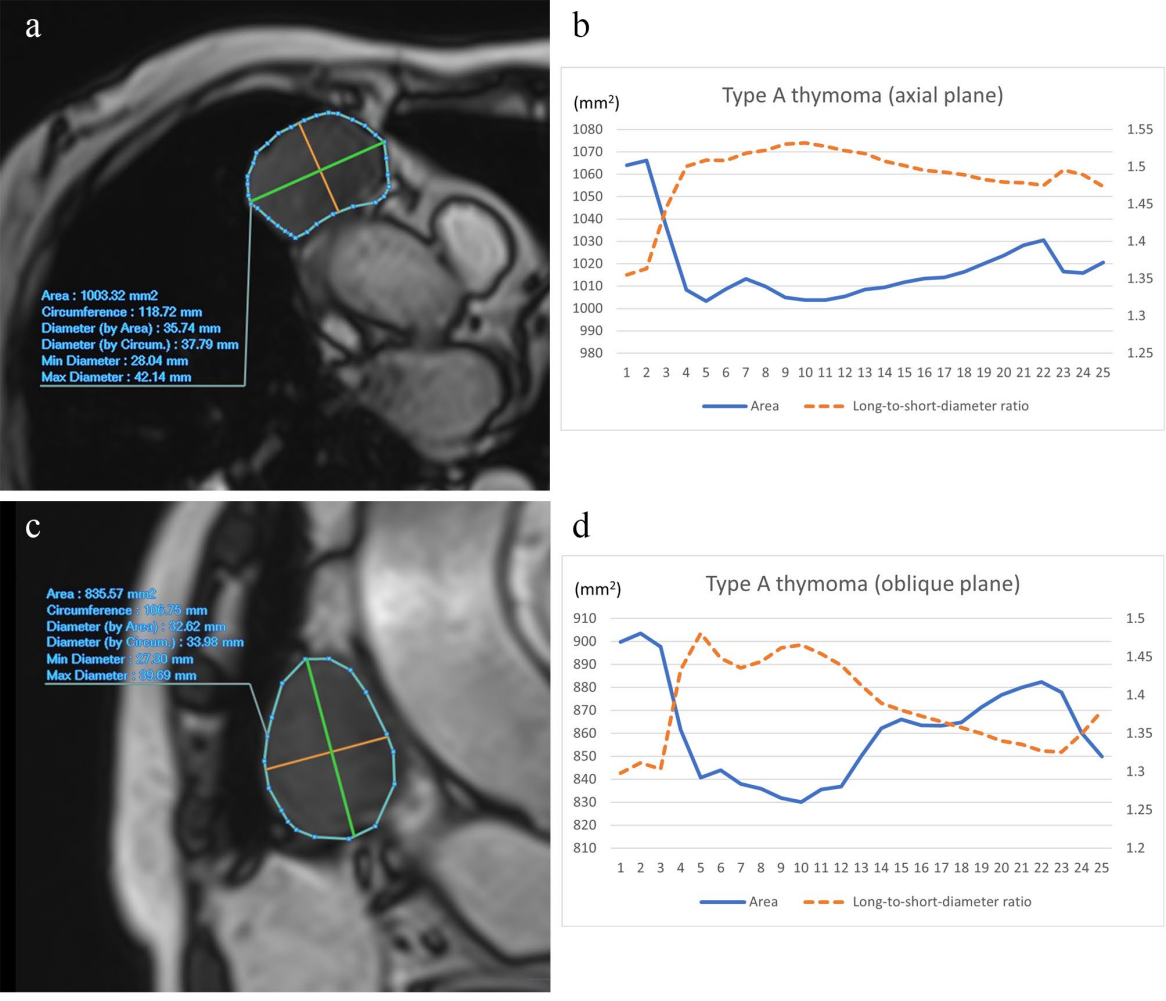
A 64-year-old woman with low-risk thymoma (type A). Axial (**a**) and oblique (**c**) cine MR images of low-risk thymoma (type A) in the right anterior mediastinum, accompanied by graphs representing the time course of area and long-to-short diameter ratio in axial (**b**) and oblique planes (**d**). In the axial plane, ∆long-to-short diameter ratio and ∆area were 10.58% and 5.89%, respectively. In the oblique plane, ∆long-to-short diameter ratio and ∆area were 10.50% and 8.14%, respectively

**Fig. 4:**
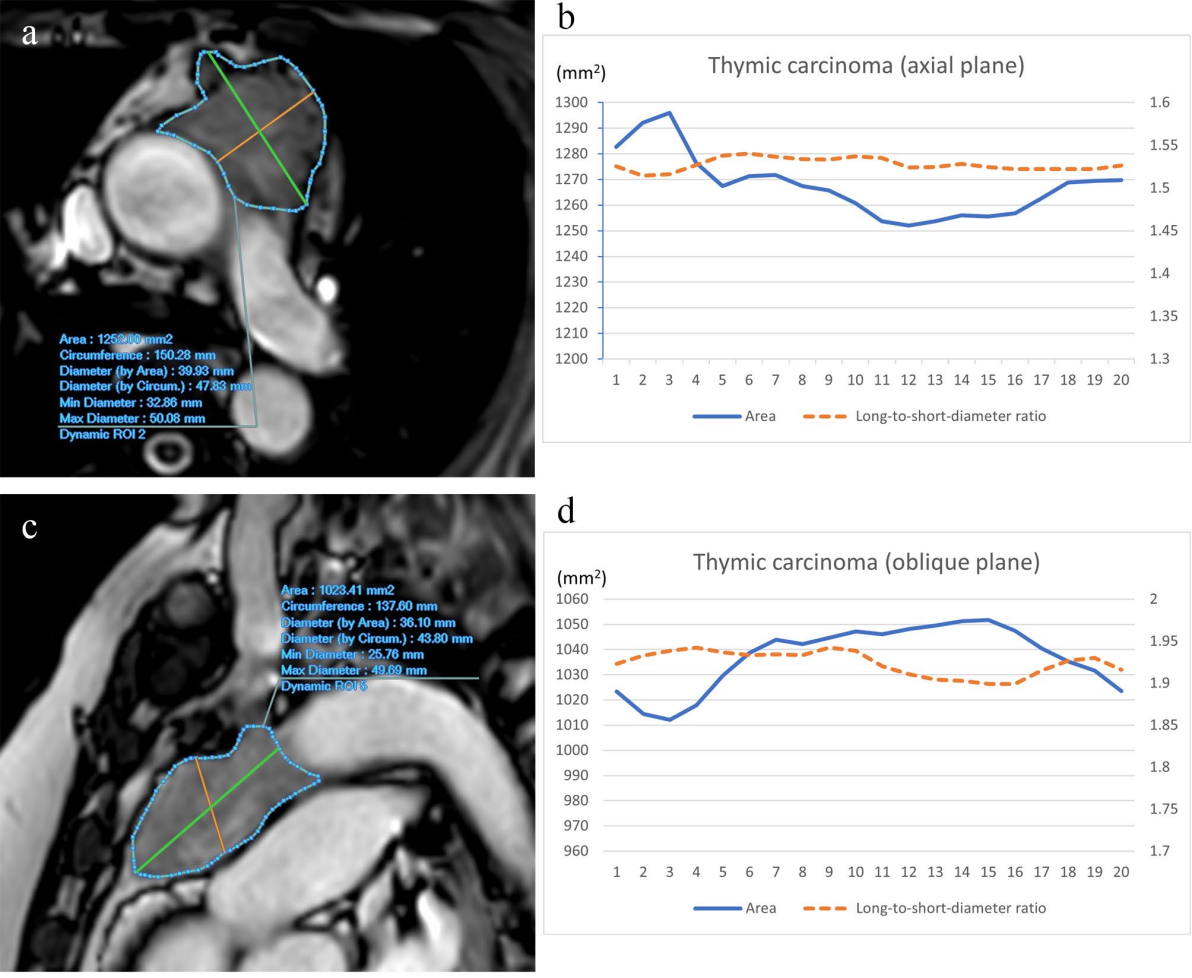
A 57-year-old woman with thymic carcinoma. Axial (**a**) and oblique (**c**) cine MR images show an irregularly shaped and ill-defined tumor in the anterior mediastinum. Graphs represent the time course of area and long-to-short diameter ratio in axial (**b**) and oblique planes (**d**). In the axial plane, ∆long-to-short diameter ratio and ∆area were 0.52% and 3.39%, respectively. In the oblique plane, ∆long-to-short diameter ratio and ∆area were 2.05% and 3.74%, respectively

Representative cases are shown in Figs. 3 and 4, and online-supplementary videos.

### Association between feature tracking parameters and cystic/necrotic changes or adjacent cardiovascular structures

Cystic/necrotic changes were present in 10 lesions (3 low-risk thymomas, 4 high-risk thymomas, and 3 thymic carcinomas), and in all cases, these changes occupied less than half of the lesion. There was no significant difference between the lesions with and without cystic/necrotic changes in ∆LSR and ∆area in both planes (all, p > 0.05).

Twenty-five lesions (3 low-risk thymomas, 17 high-risk thymomas, and 5 thymic carcinomas) predominantly contacted the aorta, 12 (4 low-risk thymomas, 5 high-risk thymomas, and 3 thymic carcinomas) predominantly contacted the pulmonary artery, and 6 (3 low-risk thymomas, 1 high-risk thymomas, and 2 thymic carcinomas) predominantly contacted the right atrium. No significant difference was observed among the predominantly contacted adjacent cardiovascular structures in ∆LSR and ∆area in both planes (all, p > 0.05).

## Discussion

The results of the present study demonstrate the feasibility of cine MRI-based feature tracking analysis for assessing cardiac-induced deformation to diagnose TETs. Values of ∆LSR and ∆area were significantly different between the TET subtypes, and those of thymic carcinomas were significantly smaller than those of thymomas. AUCs for ∆LSR and ∆area to diagnose thymic carcinomas ranged from 0.755 to 0.764. To the best of our knowledge, this potential role of cine-MR feature tracking analysis for diagnosing TETs has not been demonstrated previously.

Tissue stiffness can provide additional information in evaluating several organs [13–17], and radiological approaches such as ultrasound and MR elastographies have been developed for such evaluation [15, 16]. Measurement of liver stiffness using MR elastography is useful to predict the stage of liver fibrosis and long-term progression and outcome in chronic liver disease [15, 17]. Stiffness of the thymus in healthy children evaluated by MR elastography presented a mild negative correlation with age, height, and weight, which can be influenced by changes in the thymic epithelial space and the perivascular space [18]. Cine-MR feature tracking analysis can also provide information on tissue stiffness [8, 19–21], in which tissue deformation is inversely proportional to tissue stiffness. Cardiovascular MR feature tracking to quantify myocardial strain has been reported to be associated with ventricular stiffness [19], and cardiac-induced deformation using cine-MR tagging analysis has been used to assess liver stiffness [20, 21]. Ohashi et al. reported that cardiac-induced liver deformation evaluated by the cine-MR feature tracking technique was associated with liver function in patients with adult congenital heart disease [8]. In the present results, imaging parameters associated with cardiac-induced deformation of TETs, such as ∆LSR and ∆area obtained from cine-MR feature tracking analysis, were significantly different between the histopathological TET groups. The pathological subtypes of TETs according to the WHO classification are characterized by the content of neoplastic epithelial cells and non-neoplastic immature T cells. Typical thymomas, especially type AB, B1, and B2 thymomas, contain an abundance of immature T cells and fewer interstitial cells within the lesion [9], which can result in lower tumor stiffness compared with lymphocyte-sparse TETs such as B3 thymoma and thymic carcinomas. Therefore, the cine-MR feature tracking technique may enable the assessment of stromal conditions within a lesion and could be particularly useful for evaluating lesions in which the stromal features vary by histological subtype, such as TETs.

Evaluation of lesion stiffness was reported to be useful for differentiation between benign and malignant lesions [22–24]. Malignant tumors tend to have an abundant extracellular matrix, and increased vascularity and interstitial pressure [25]. These characteristics, along with increased cellularity, may cause increased stiffness. In addition, intratumoral fibrosis is highly associated with cancer [26], which can result in increased lesion stiffness compared with benign lesions. In organs such as breast, pancreas, prostate, and lymph nodes, malignant lesions were reported to be stiffer than benign lesions or adjacent parenchymal organ tissue on ultrasound elastography [22–24, 27]. In the thoracic space, US elastography demonstrated that malignant pleural-based masses were also stiffer than benign masses [28]. A preliminary study focused on MR elastography demonstrated that the stiffness level of thymic carcinoma had a tendency to be higher than that of thymoma and lymphoma [29]. In the present results, ∆LSR and ∆area values of thymic carcinomas were significantly lower than those of thymomas, which suggests that the stiffness of thymic carcinoma is higher than that of thymomas, possibly due to the abundant fibrous tissues in these lesions. Cine MRI is a commonly used MR sequence that can be performed on almost any MR device without additional expensive functions. Therefore, it is clinically useful to be able to predict histopathological characteristics by evaluating cardiovascular-induced deformation of TETs at the same time as evaluating cardiovascular invasion [4–6].

There are limitations in the present study. First, we included only a small number of patients with TET from a single institute, which limited the statistical power and universality of the study. In addition, it was a retrospective study that may have been subject to selection bias due to the unbalanced number of patients for each TET subtype. We believe that our pilot study will encourage future research and that a well-designed prospective study with a large number of cases is needed to confirm our findings. Second, cardiac-induced deformation of TETs can be affected by the stiffness of the adjacent cardiovascular structures and by blood pressure. Further research is needed to clarify the relationship between the state of adjacent structures and cardiac-induced deformation.

## Conclusion

The present results suggest that cardiac-induced deformation of TETs evaluated using the cine-MR-based feature tracking technique may reflect differences in histopathological characteristics among TETs. Evaluation of morphological deformation using cine MRI can help diagnose histopathological subtypes of TET and identify thymic carcinomas preoperatively.

## Electronic supplementary material

Below is the link to the electronic supplementary material.

Supplementary Material 1

Supplementary Material 2

Supplementary Material 3

Supplementary Material 4

Supplementary Material 5

## Data Availability

The datasets of current study are available from the corresponding author on reasonable request.

## Abbreviations

TET: Thymic epithelial tumor
WHO: World Health Organization
MRI: Magnetic resonance imaging
bSSFP: Balanced steady-state free precession
LD: Long diameter
SD: Short diameter
LSR: Long-to-short diameter ratio
LA: Lesion area
∆LSR: Change in long-to-short diameter ratio between the two phases when lesion areas were maximal and minimal
∆area: Change in lesion area between the two phases when lesion areas were maximal and minimal
ROC: Receiver-operating characteristic
AUC: Area under the ROC curve
ICC: Intraclass correlation coefficient
CI: Confidential interval
