# Supplementary material for: Cine MR feature tracking analysis for diagnosing thymic epithelial tumors: a feasibility study
Source: Cancer Imaging. 2023 May 1;23:42. doi: 10.1186/s40644-023-00560-z (PMC10150474; doi:10.1186/s40644-023-00560-z)
Supplement: Supplementary file 5 — Supplementary Material 5 [file 40644_2023_560_MOESM5_ESM.docx]

| **Online supplementary table. Intra-observer agreements** | |
| --- | --- |
| Parameters | ICC (95%CI) |
| Axial plane |  |
| Lesion diameter on maximal lesion area | 0.997 (0.994–0.998) |
| Lesion diameter on minimal lesion area | 0.998 (0.996–0.999) |
| Short diameter on maximal lesion area | 0.997 (0.994–0.998) |
| Short diameter on minimal lesion area | 0.998 (0.995–0.999) |
| Long-to-short diameter ratio on maximal lesion area | 0.983 (0.969–0.991) |
| Long-to-short diameter ratio on minimal lesion area | 0.986 (0.974–0.992) |
| Lesion area on maximal lesion area | 0.999 (0.998–1.000) |
| Lesion area on minimal lesion area | 0.999 (0.998–1.000) |
| Change in long-to-short diameter ratio | 0.954 (0.917–0.975) |
| Change in lesion area | 0.995 (0.990–0.997) |
| Oblique plane |  |
| Lesion diameter on maximal lesion area | 0.990 (0.982–0.995) |
| Lesion diameter on minimal lesion area | 0.993 (0.988–0.996) |
| Short diameter on maximal lesion area | 0.991 (0.983–0.995) |
| Short diameter on minimal lesion area | 0.987 (0.977–0.993) |
| Long-to-short diameter ratio on maximal lesion area | 0.982 (0.966–0.990) |
| Long-to-short diameter ratio on minimal lesion area | 0.968 (0.942–0.983) |
| Lesion area on maximal lesion area | 0.997 (0.995–0.998) |
| Lesion area on minimal lesion area | 0.998 (0.996–0.999) |
| Change in long-to-short diameter ratio | 0.742 (0.570–0.851) |
| Change in lesion area | 0.893 (0.811–0.941) |
